# Is Spirituality Included in Spanish Language Pain Assessment Scales? A Scoping Review of Instruments Available

**DOI:** 10.1007/s10943-025-02343-1

**Published:** 2025-06-05

**Authors:** Rocio de Diego-Cordero, Emilio Martin-Palomino, Alicia Aranda-Jerez, Jose Miguel Perez-Jimenez, Giancarlo Lucchetti

**Affiliations:** 1 Department of Nursing, Schools of Nursing, Physiotherapy and Podiatry, University of Seville, C/ Avenzoar 6, 41009 Seville, Spain; 2 Research Group CTS1149: Integral and Sustainable Health: Bio-Psycho-Social, Cultural and Spiritual Approach for Human Development, Seville, Spain; 3 Anaesthesiology and Resuscitation Clinical Management Unit, University Hospital Virgen Macarena, Seville, Spain; 4 School of Medicine, Federal University of Juiz de Fora, Juiz de Fora, MG 36047-000 Brazil

**Keywords:** Grief, Grief dimension, Spirituality, Life course perspective, Hope, Health professional

## Abstract

The subjective and biopsychosocial–spiritual nature of pain makes its assessment difficult. Although multidimensional scales exist, few pain instruments have included spiritual aspects in their development, so our purpose is to investigate the inclusion of spirituality in pain assessment scales available in Spanish. A review of the scientific literature was carried out in the PubMed, SCOPUS and Web of Science databases. To make the search more exhaustive, Internet databases were searched, and the Spanish Pain Society was consulted. For each instrument, items were rated and classified according to a broader (i.e., family, nature, transcendence, meaning) or narrower (i.e., deity or higher power) definition of spirituality. Out of a total of 838 items searched in the databases, 8 multidimensional pain scales were found in Spanish, and 5 included items related to spirituality and/or religiosity. Most of the scales do not focus directly on the assessment of spiritual pain. Although the Coping with Chronic Pain Questionnaire has clear items related to prayer, God, faith and seeking religious leaders, the other scales only touch on spiritual definitions and most of these would lead to “contaminated” results due to poor design. It seems that these scales were not originally designed to assess the spiritual dimension of pain but, instead, have some existential items that are linked to a broader definition of spirituality, such as hope, suffering, emptiness and meaning. The present review has revealed that most pain scales available in Spanish were not originally designed to investigate spiritual pain, assessing indirectly items related to spirituality. These findings highlight that, although spirituality is an important aspect of pain, its assessment is still scarce in clinical practice and research. Future instruments should include a more holistic view of pain, which includes aspects related to spirituality and religiousness.

## Introduction

Pain can be defined as an unpleasant sensory and emotional experience associated with, or expressed in terms of, present or potential harm. Since pain is a self-precepted sensation, it is unique to each individual and plays an adaptive role (Raja et al., 2020). Although pain is considered a physical experience, new guidelines and definitions are broadening the definitions of pain, aiming to include other biopsychosocial dimensions (Flor et al., 2023).

Among several dimensions of pain, cultural aspects are included as a factor associated with the perception and the experience of pain. Within this context, spirituality appears as an important, but commonly overlooked factor associated with pain (Badanta et al., 2022). The term spirituality derives from the Latin "*spiritus*," which refers to "breath" or "wind." It represents the life force that “animates” a person and permeates all aspects of his or her existence (Potter et al., 2023).

The scientific definition of spirituality is not consensual and there are currently different understandings for its meaning. Some authors use a narrower definition of spirituality, referring to a deity, sacred or higher power, while others believe spirituality should be viewed as a broader concept, which encompasses the search for the “significant,” “sacred,” but also which holds ultimate meaning or purpose (e.g., relationships with others, the transcendent, nature or the self) (Steinhauser et al., 2017).

Therefore, spiritual suffering is linked to pain, and addressing it provides optimal health care (Costeira, et al., 2024). Recent studies have supported the role of spirituality on the provision of a more holistic health care, which have endorsed several medical and nursing guidelines (Murgia, et al., 2020). Similarly, understanding, assessing and alleviating the spiritual suffering associated with pain represent a fundamental challenge both in clinical practice and in pain research (Noe-Steinmüller et al., 2024).

The inclusion of a more comprehensive model of pain is crucial to establish an approach to care that is both humane and supported by science, facilitating more effective responses to the challenges posed by serious and terminal illnesses.

Among the various nursing theories that address the spiritual aspect of patient care, the Theory of Human Caring stands out (Aghaei, 2020). This theory highlights the importance of care above a simple medical cure, recognizing the mysterious aspects of life and valuing the spiritual dimension and the “inner power” in the process of care, where the health professional works as an active partner.

Is argued that it is crucial for health professionals to consider the spiritual dimension when caring for patients facing serious illness to mitigate patients’ spiritual suffering. In addition, health professionals may find it beneficial to base their caring practice on a theory such as Jean Watson's, which addresses both the physical and non-physical aspects of the patient, recognizing the connection between mind, body and spirit (Evangelista, 2022).

Despite these new models of care and new ways to assess pain, the pain assessment scales still overlook spirituality as a detrimental dimension of pain. Pain assessment scales are instruments used by health professionals to assess various aspects of a patient's condition, which are fundamental for clinical practice as they allow continuous assessment and effective communication with the patient, guiding decision-making and serving to monitor therapy.

Since the way people experience pain is complex and subjective, direct patient reporting is still the most accurate and reliable way of knowing about the existence and grade the intensity of pain (Karcioglu, 2018). The integration of a pain education program leads to greater patient knowledge in the spheres of pain, positively impacting their health (Sidiq, et al., 2024). This rule applies to people of all age groups, even if they have cognitive problems or difficulties in communication (Karcioglu, 2018).

However, this subjective and multidimensional nature of pain makes its assessment particularly challenging, and the use of validated scales is necessary. These scales, designed to measure and/or communicate the patient's pain, can be classified into two groups: unidimensional and multidimensional scales. It is important to note that unidimensional scales only assess pain intensity and do not provide a complete picture of the patient's experience, such as the case of some of the most used scales in the clinical setting such as the numerical rating scale (NRS), the visual analogue scale (VAS) and the verbal rating/description scale (VRS/VDS) (Karcioglu, 2018).

To be in line with this new comprehensive view of pain, multidimensional scales are composed of different domains, including the spiritual domain of pain. Therefore, understanding and exploring which scales have the spiritual aspect of pain included in their development is crucial to health professionals and could help managers and educators to choose the best pain tool for training and for assessing patients (Evangelista, 2022).

This scoping review aims to investigate how spirituality is incorporated in the pain assessment scales available in the Spanish language, as well as separate if this inclusion uses a broader or narrower definition of spirituality.

## Methods

A scoping review of the literature was carried out. As defined by the Joanna Briggs Institute, this is “a type of evidence synthesis that aims to systematically identify and map the breadth of evidence available on a particular topic, field, concept or issue, often irrespective of source (i.e., primary research, reviews, non-empirical evidence) within or across contexts. Scoping reviews can clarify key concepts/definitions in the literature and identify key characteristics or factors related to a concept, including those related to methodological research” (Peters, et al., 2020).

To be included, references should have instruments/scales that assess pain, that were developed, translated and/or adapted into the Spanish language and that were multidimensional, i.e., that included several dimensions of pain. Unidimensional scales such as the visual analogue scale were not included, since they have the primary objective of assessing analgesia and, therefore, do not include other dimensions such as psychological, social or spiritual aspects. Opinion articles, comments and editorials were also excluded.

The literature search was carried out in March 2024 including the following databases: PubMed, Scopus and Web of Science. The strategy to be followed was based on the DeCS/MeSH health science descriptors: "pain scale," "pain rating scale," "pain assessment," spiritual, religion and faith, in combination with the Boolean operators AND and OR. A librarian from the University of Seville was consulted for this strategy, which can be visualized in Table 1.

**Table 1:** Search strategy

Database	Strategy	Articles found	Total of articles
PUBMED	("pain scale" OR "pain rating scale" OR "pain measurement") AND (spiritual OR religion OR faith OR prayer)	319	838
WOS	("pain scale*" OR "pain rating scale*" OR "pain measurement*") AND (spiritu* OR religi* OR faith OR prayer)	273	
SCOPUS		246	

Once the search was completed, all the references were exported to Zotero. Zotero is a bibliographic reference management software that allows you to collect, organize, cite and share bibliographic references from books, journal articles, websites and other types of documents.

Aiming to have a more comprehensive search, Google Scholar and Internet searches were also consulted, using the term “Pain Scales” and “Spanish” or “Spain,” so that this strategy would reflect all pain scales, both unidimensional and multidimensional.

Precautions were taken when selecting these online sources. Only sources with academic or institutional background were used, and unverified information was avoided. Likewise, at least two researchers have assessed these references before being included in the review.

Finally, after the database searches, authors also contacted representatives from Spanish pain organizations (i.e., Spanish Pain Society and the Andalusian Pain Society) aiming to include scales that were not found in our searches, but that are used and/or known by pain experts in clinical practice and research. This contact was carried out first by phone, where the objectives of the study were presented to a representative, and then, an email was sent reminding this representative to provide possible eligible instruments/scales.

Assessment of the adherence to reporting guidelines was also investigated, using STROBE for observational studies and Standards for Reporting Qualitative Research (SRQR) guidelines for qualitative studies, when applicable.

Searches and the assessment of eligible references were performed independently by two researchers. Titles and abstracts were read to check the eligibility, and after that, articles were read in full, and the scales were consulted item by item to evaluate whether there are items related to the spiritual aspects of pain. For this matter, we have evaluated whether this inclusion was for a broader (i.e., meaning or purpose: relationships with others, the transcendent, nature or the self) or narrower (i.e., deity, sacred or higher power) definition of spirituality as presented in the introduction section. Finally, researchers assessed whether the items could be considered “contaminated.” In other words, items with tautological problems (i.e., overlapped with indicators of good mental health, psychological well-being and social connections), although included in the review, were marked as “contaminated,” aiming to provide a clear perspective of the tautological problems to the readers (Koenig & Carey, 2024, 2025).

## Results

**Fig. 1:**
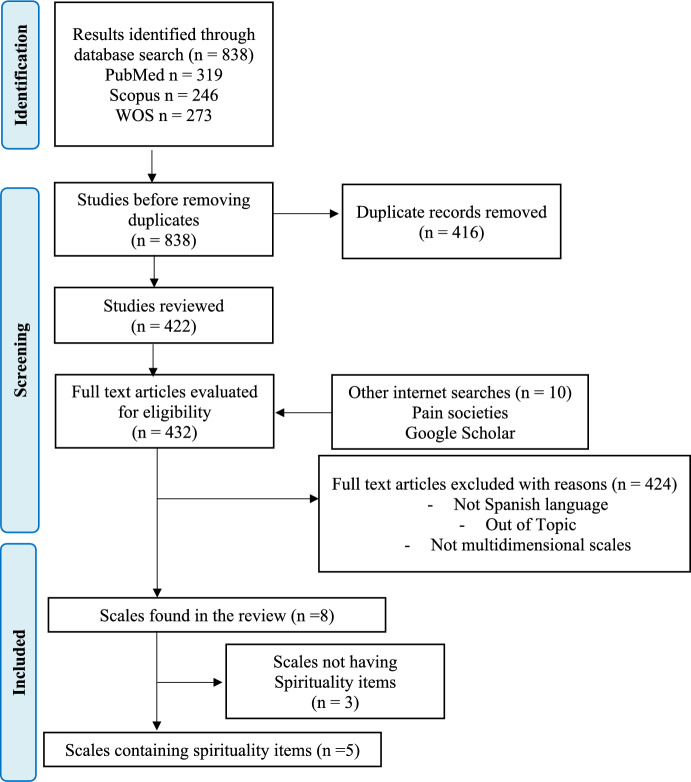
Flow diagram

A total of 838 articles were found: 319 in PubMed, 273 in Web of Science and 246 in SCOPUS. After removing duplicates, a total of 422 documents remained. A total of 10 articles were found through other searches (i.e., Google Scholar searches [*n* = 8] and consultations with the Spanish Pain Society [*n* = 2]). Figure 1 shows the flow diagram with the reasons for exclusion. For the final analysis, 424 articles were excluded, totaling 8 articles with instruments that were analyzed.

After analyzing the multidimensional pain scales, the focus was placed on the spirituality items, as explained below. Among these instruments, 3 were excluded because did not have any spirituality item (i.e., Pain Self-Efficacy Questionnaire, Edmonton Symptom Screening Questionnaire, Brief Pain Inventory) and 5 scales were included (i.e., Chronic Pain Coping Questionnaire, West Haven Yale Multidimensional Pain Inventory, Pain Catastrophizing Scale, Spanish Pain Questionnaire and Psychological Pain Scale).

Table 2 presents in detail the scales assessed and the items related to spirituality or religion. Among all instruments, the Chronic Pain Coping Questionnaire was the one with most items related to the concept of spirituality and religion. This instrument seems to be designed to include religious and spiritual beliefs, and this association is clearly linked to the definition of religion and spirituality. Likewise, this scale was the only associated with religious aspect. (Four items were directly related to religions such as prayer and seeking a religious leader.)

**Table 2:** Multidimensional pain scales included in the review and the items related to spirituality or religion

Scale	Item	Dimension	Definition used*	Does it have a possible contaminated item?**	Methodological Quality*** STROBE
Chronic Pain Coping Questionnaire	1. I pray for my pains to disappear	Religion	organized system of beliefs, practices and symbols	No	19/22
	2. I pray for strength and guidance regarding the problem	Religion	organized system of beliefs, practices and symbols	No	
	3. I pray to be healed	Religion	organized system of beliefs, practices and symbols	No	
	4. I use faith to alleviate my pains	Spirituality	relationship to the sacred or transcendent, which may (or may not) lead to or arise from the development of religious rituals	No	
	5. I ask God to relieve me of my pains	Spirituality	relationship to the sacred or transcendent, which may (or may not) lead to or arise from the development of religious rituals	No	
	6. When I am in pain, I try to talk to someone and tell them what is happening to me. This helps me cope with it	Spirituality	persons seek ultimate meaning, purpose … experience relationship to self, family, others,	Yes	
	22. I think I must have strength and not falter	Spirituality	understanding answers to ultimate questions about life, about meaning OR seek ultimate meaning, purpose	Yes	
	29. I talk to a professional (doctor, psychologist, priest, etc.) about the problem to help me cope with it	Religion	organized system of beliefs, practices and symbols (e.g., priest)	No	
West Haven Yale Multidimensional Pain Inventory	12. How much suffering do you experience because of your pain?	Spirituality	understanding answers to ultimate questions about life, about meaning OR seek ultimate meaning, purpose	Yes	20/22
Pain Catastrophizing Scale	2. I feel like I can't go on anymore because of the pain	Spirituality	understanding answers to ultimate questions about life, about meaning OR seek ultimate meaning, purpose	Yes	20/22
	5. I feel like I can't endure the pain any longer	Spirituality	understanding answers to ultimate questions about life, about meaning OR seek ultimate meaning, purpose	Yes	
Pain Self-Efficacy Questionnaire	No items				18/22
Spanish Pain Questionnaire	Fear: 1. Fearful 2. Frightful 3.Terrifying	Spirituality	understanding answers to ultimate questions about life, about meaning OR seek ultimate meaning, purpose	Yes	19/22
	Punishment 1.Punishing 2. Grueling 3. Cruel 4. Vicious 5. Killing	Spirituality	understanding answers to ultimate questions about life, about meaning OR seek ultimate meaning, purpose	Yes	
	Affective: 1. Nagging 2.Nauseating 3. Agonizing 4. Dreadful 5. Torturing	Spirituality	understanding answers to ultimate questions about life, about meaning OR seek ultimate meaning, purpose	Yes	
Psychological Pain Scale	5. My pain makes my life dark	Spirituality	understanding answers to ultimate questions about life, about meaning OR seek ultimate meaning, purpose	Yes	19/22
	8. It hurts because I feel empty	Spirituality	understanding answers to ultimate questions about life, about meaning OR seek ultimate meaning, purpose	Yes	
	9. My soul hurts	Spirituality	understanding answers to ultimate questions about life, about meaning OR seek ultimate meaning, purpose	No	
Edmonton Symptom Screening Questionnaire	No items: there is a study incorporating a spiritual pain item in the Edmonton Symptom Questionnaire. But this was not examined in the Spanish reality				18/22
Brief Pain Inventory	No items				19/22

*Definitions used: Religion (Koenig) is an organized system of beliefs, practices and symbols designed (a) to facilitate closeness to the transcendent and (b) to foster an understanding of one's relationship and responsibility to others in living together in a community. Spirituality (Koenig) is the personal quest for understanding answers to ultimate questions about life, about meaning and about relationship to the sacred or transcendent, which may (or may not) lead to or arise from the development of religious rituals and the formation of community – REFERENCE: Koenig HG. Research on religion, spirituality and mental health: a review. Can J Psychiatry. 2009 May;54(5):283–91. https://doi.org/10.1177/070674370905400502. PMID: 19,497,160

Spirituality (Puchalski) is a dynamic and intrinsic aspect of humanity through which persons seek ultimate meaning, purpose and transcendence, and experience relationship to self, family, others, community, society, nature and the significant or sacred. – REFERENCE: Puchalski CM, Vitillo R, Hull SK, Reller N. Improving the spiritual dimension of whole person care: reaching national and international consensus. J Palliat Med. 2014 Jun;17(6):642–56. https://doi.org/10.1089/jpm.2014.9427. Epub 2014 May 19. PMID: 24,842,136; PMCID: PMC4038982

**Contaminated items were assessed using previous JORH publications. Some spirituality items were considered contaminated if they were overlapped with indicators of good mental health, psychological well-being and social connections, providing tautological problems. REFERENCES: Koenig, H.G., & Carey, L.B. (2024). Religion, Spirituality and Health Research: Warning of Contaminated Scales. Journal of Religion and Health, 63 (5). And Koenig, H.G. & Carey, L.B. (2025). Approaches for Analyzing the Relationship Between Spirituality and Health Using Measures Contaminated with Indicators of Mental and Social Health. Journal of Religion and Health 64 (1)

***1 = recommendation contained in the study, 0 = recommendation not included NA = not applicable

Other instruments, such as Chronic Pain Coping Questionnaire, West Haven Yale Multidimensional Pain Inventory, Pain Catastrophizing Scale, Spanish Pain Questionnaire and Psychological Pain Scale had existential issues such as suffering, fear, punishment and emptiness, which were associated to the concept of spirituality, as relates to “understanding answers to ultimate questions about life, about meaning” or “seek ultimate meaning, purpose.” Although these items are related to spiritual and existential issues, they could be considered “contaminated” items, since these are overlapped with indicators of good mental health, psychological well-being and social connections, providing tautological problems. Only three spirituality items in our review “My soul hurts” (from the Psychological Pain Scale) and “I ask God to relieve me of my pains” and “I use faith to alleviate my pains” from the Chronic Pain Coping Questionnaire were considered uncontaminated items according to the work of Koenig and Carey (Koenig & Carey, 2024, 2025).

Finally, 3 scales did not have any relationship to spirituality, i.e., Pain Self-Efficacy Questionnaire, Edmonton Symptom Screening Questionnaire, Brief Pain Inventory. It is important to highlight that there is a study incorporating a spiritual pain item in the Edmonton Symptom Questionnaire. But this was not examined in the Spanish reality.

Quality assessment revealed that most studies had appropriate research questions, clearly defined populations, good response rates, appropriate exposure and outcome variables, and control of confounding factors. In the assessment of adherence to reporting guidelines, all articles included in this review were considered to be of high or medium quality for observational studies (STROBE). Following assessment using the Standards for Reporting Qualitative Research (SRQR) guideline, no articles were rejected, and studies met most of the items. However, the most frequently non-compliant item related to researcher characteristics and techniques to increase reliability.

## Discussion

The present scoping review aimed to analyze pain scales that incorporate spirituality into their items. Overall, the findings of this study, within the biopsychosocial–spiritual perspective of pain, corroborate to the influence of spirituality in different aspects of pain. To this end, multidimensional scales for pain assessment have been analyzed, reflecting that spirituality and religion could be assessed in different ways, which includes religious practices, spiritual beliefs, faith, hope, suffering, emptiness and meaning.

Although there is a perception that only the biological dimension of pain should be considered, recent evidence is moving to a more holistic and integrative approach, highlighting that all dimensions should be considered and incorporated into the assessment of pain. A recent article, published in one of the most important journals related to pain, has advocated the “inclusion of spiritual factors as an important component in the assessment and treatment of pain” (Siddall et al., 2015). In this context, the present review aims to shed a light on this topic, discussing the role of spirituality and its incorporation into pain assessment instruments.

Our first finding here is that most scales do not have a direct approach to assess spiritual pain. Although the Chronic Pain Coping Questionnaire has clear items related to prayer, God, faith and religious leader seeking, the other scales only permeate spiritual definitions through items related to suffering and meaning. It seems that these scales were not originally designed to assess the spiritual dimension of pain, but they some existential items that are linked to a broader definition of spirituality.

Regarding the approach to spirituality from the perspective of hope, the pain scales included in this study indicate that hope is an important variable for coping with pain. Previous studies have pointed out that patients with higher levels of optimism and hope, associated with spirituality-based coping resources, correlated with lower rates of complications, as well as a reduction in anxiety, stress and depression. Furthermore, spirituality and hope are related to lower levels of cytokines and other inflammatory markers, serving as a protective factor against pain (Negré et al., 2023).

On the other hand, distress and spiritual crisis are proportionate to pain; this occurs when individuals are unable to find sources of hope, resulting in a detrimental effect on physical and mental health (Anandarajah & Hight 2021). Another study indicates that patients with high levels of spirituality tend to develop strategies to care for and positively cope with stressful situations such as pain. In contrast, patients with lower levels of spirituality cope with their health conditions ineffectively and are at risk of becoming depressed (hopelessness) (Núñez et al., 2012).

Consequently, spirituality provides a sense of meaning to life events, helps in coping with pain and offers comfort and hope in the face of problematic or poor prognosis ailments (Klimasiński, 2021). When the experience of pain is accompanied by hope, the patient focuses their energy on restoring health and well-being (Ottaviani et al., 2014). This study highlights that when assessing the pain of patients using scales with a spiritual dimension, hope will determine the perception of pain, with the patient's hopelessness being a risk factor.

Regarding the meaning of life as a tool of spirituality in pain scales, previous studies suggest that finding meaning in life is fundamental for recovering from stressful experiences, such as pain, reducing posttraumatic symptoms and creating positive change after a traumatic experience (Mehnert, 2006). Furthermore, finding a sense of purpose in life increases the likelihood of understanding pain, allowing for the development of better strategies to cope with it resilience strategies that can ensure multidimensional care based on the biopsychosocial aspects of the whole being (Mehnert, 2006; Medeiros et al., 2020).

Another study indicates that when individuals feel their lives lack purpose, they experience profound frustration that can lead to despair. This connection between inner emptiness and hopelessness is intimate; both states affect mood, motivation, feelings and future expectations. They manifest as apathy, fatigue, lack of enthusiasm, a sense that life lacks meaning, belief in an inevitable fate and a loss of direction, exacerbating the pain they endure (Moura et al., 2020).

Regarding the affective–sensory dimension of spirituality in pain scales, this study demonstrates that understanding emotions and feelings as adaptive responses both positive and negative means that these affective responses proportionally influence coping and how the patient perceives pain. The literature shows that positive affectivity has traditionally been linked to health, and there is a direct connection between the two. In fact, the development of positive feelings and emotions plays a preventive role concerning illness, and especially pain (Garcia-Alandete et al., 2009).

Another study highlights that spirituality not only interacts with emotions and feelings but also cultivates them, serving as a very useful tool in managing pain (Cantus, 2011). The painful sensation is accompanied by anxiety, depression, fear, distress, etc., which are stressors that exacerbate the affective–emotional dimension of the patient, impacting their health (Mariño, 2020).

Despite the inclusion of existential and spiritual aspects among some pain assessments in our review, it is important to highlight that all items, except for 3 items available in two scales (i.e., Psychological Pain Scale and Chronic Pain Coping Questionnaire) had uncontaminated items based on the research of Koenig and Carey (Koenig & Carey, 2024, 2025).This could be considered a problem, since tautological problems may arise with such an approach, particularly when using these scales for research. Future instruments should consider items that are not overlapped with well-being and mental health, providing more accurate data for the research on the spiritual aspect of pain.

### Strengths and Limitations

This study has important strengths. This scoping review has allowed us to compile and identify the importance of spirituality in multidimensional pain scales as a variable that needs to be worked on. And it has been a tool to identify knowledge gaps and new research questions. Also, other strength of conducting a scoping review include it’s that could serve as a valuable tool for synthesizing information across various studies, there by guiding future research directions and policy implications.

However, this study has several limitations that should be taken into consideration. First, although additional Internet and Google Scholar searches were carried out, the fact that the structured search relied in only three databases (PubMed, Scopus and WOS) prevented access to all the articles that could have been produced in relation to the research question posed, in addition to the impossibility of accessing the full text of some articles, which has made the search and selection of these difficult.

Second, only pain organizations in Spain were consulted. It is possible that other Spanish-speaking countries could be using other pain scales that were not captured in our review. Finally, scales were only assessed into the Spanish language, and it is possible that other pain scales for the English language may have the incorporation of spiritual or religious items.

### Implications for Clinical Practice

The present review highlighted the challenges on finding pain scales that consider the spiritual aspect, starting from the basis of pain as a biopsychosocial concept. This difficulty was related to the lack of clinical application of a more integrative approach, i.e., multidimensional pain scales with spirituality items are not commonly used because a holistic pain care is not applied; therefore, unidimensional scales such as the visual analogue scale are applied considering only analgesia as the primary goal of care.

The hope presented in multidimensional grief scales can change how each person interprets the situation. Those who retain hope may feel some relief even in the midst of intense pain, while those who lack hope may feel overwhelmed by despair. It is essential to understand that hope does not always make the pain go away, but it can provide comfort and strength to cope with it. Ultimately, hope reminds us that there is the possibility of change, of healing and of finding meaning even in the most difficult times. In this sense, hope not only accompanies pain, but also transforms it, offering a glimmer of light in the midst of darkness.

In the midst of the most intense pain, the meaning of life takes on a special significance as it becomes a constant search for purpose and meaning. While some feel hopeless, others see pain as an opportunity to grow, learn and connect with themselves on a deeper level. Suffering challenges us to reconsider our priorities, to appreciate meaningful relationships and to find deeper meaning in our experiences. The meaning of life in these circumstances lies in our ability to find hope, gratitude and meaning in even the harshest adversity, thus turning pain into a source of personal growth and strength, or as a tool for coping with pain.

In assessing pain, it is essential to take into account its emotional impact, as this is an integral part of the experience of suffering. It is not enough to measure physical intensity alone; we must also recognize how it affects people emotionally. This involves considering feelings such as anxiety, depression, fear and distress, which can accompany physical pain and affect an individual's quality of life and emotional well-being. Therefore, for a complete and accurate assessment, as well as to provide compassionate and effective treatment, it is crucial that the affective–emotional dimension is integrated into pain scales and addressed clinically.

However, unfortunately, there is little training in the spiritual approach to pain on the part of health professionals, either in the assessment or in the implementation of a care plan. Once the patient has undergone a pain assessment using scales or questionnaires that consider the spiritual aspect, nursing diagnoses must be established that cover pain, such as [00132] acute pain, [00133] chronic pain, [00225] chronic pain syndrome or [00256] labor pain.

Making nursing diagnoses is essential in patient care, as it provides a sound basis for planning and delivering personalized and effective care. In the case of pain, nursing diagnosis is vital to identify, assess and treat this subjective sensation effectively.

## Conclusions

The present review has revealed that most pain scales were not originally designed to investigate spiritual pain, assessing indirectly items related to spirituality such as suffering, hope and meaning. Only one scale was found assessing religious aspects that could be important as a mechanism of coping for patients as well. These findings highlight that, although spirituality is an important aspect of pain, its assessment is still scarce in clinical practice and research. Future instruments should include a more holistic view of pain, which includes aspects related to spirituality and religiousness but ensure to avoid potential contamination. 

Care plans are not applied in which the patient's spirituality is worked on. The approach to pain has always been relegated to the application of analgesics, without addressing the biopsychosocial concept of pain, thus enhancing the spiritual aspect.

## Appendix

See Table 2.
